# A291 CLINICAL OUTCOMES IN PATIENTS WITH MODERATE TO SEVERE PANCREATITIS AND PANCREATIC COLLECTIONS: A RETROSPECTIVE COHORT STUDY

**DOI:** 10.1093/jcag/gwac036.291

**Published:** 2023-03-07

**Authors:** A Kundra, P Mundra, S Srichandramohan, C Sypkes, N Calo

**Affiliations:** 1 Department of Gastroenterology, University of Ottawa, Ottawa; 2 McMaster University, Hamilton; 3 University of Ottawa, Ottawa; 4 Department of Medicine, University of British Columbia, Vancouver, Canada

## Abstract

**Background:**

Necrotizing pancreatitis occurs in approximately 20% of patients with pancreatitis and is associated with significant morbidity and mortality. Drainage of pancreatic collections is critical when there is associated infection but is also indicated in patients with biliary or gastric outlet obstruction or symptoms. Drainage strategies include percutaneous, endoscopic ultrasound (EUS) guided, and surgical. Data suggests that a step-up approach starting with EUS or percutaneous drainage is associated with lower mortality and morbidity at 3-6 months in comparison to surgery. Little is known about risk factors for pancreatic necrosis and long-term outcomes in patients with pancreatic collections, specifically those with necrotic collections.

**Purpose:**

To improve knowledge about risk factors and long-term outcomes in patients with pancreatic collections. The primary objective was to compare mortality between patients with and without early necrotic collections. Secondary outcomes included ICU stay, recurrent collections and repeated interventions during follow-up.

**Method:**

This was a retrospective cohort study of consecutive adults (> 18 years) with moderate to severe acute pancreatitis as per the Atlanta Criteria, admitted to a tertiary care centre in Ontario between January 2002 and January 2019 with radiological evidence of early pancreatic collections. Patients were identified using administrative codes and imaging reports from the Hospital’s data warehouse. Descriptive statistics were used. Comparisons between groups were made with chi-square and logistic regression for categorical variables.

**Result(s):**

723 patients were identified and 276 were included in this report. The mean age was 54.5 years (SD 16) and 109 (39.5%) were females. The most common comorbidities were diabetes (33.3%), hypertension (38.4%) and obesity (14.8%). The most common pancreatitis etiologies were biliary (34.4%) alcohol misuse (17.0%) and post-ERCP pancreatitis (7.1%). Eighty-five (30.8%) patients were diagnosed with early necrotic collections and 53 (19.2%) with peri-pancreatic fluid collections during the initial 30 days of follow-up. Drainage was performed in 100 (36.2%) patients. Patients with necrotic collections were most likely to be obese (29.4% vs. 8.4%, P <0.001), have biliary pancreatitis (50.6% vs. 27.2%), require drainage of the collection (63.5% vs. 24.1%, P < 0.001), ICU stay (62.3% vs. 33.5%, P <0.001) and develop new collections during follow up (0% vs. 20.1%, P=0.04). Sixty-one (22.1%) patients died during a median follow up of 727 days (IQR 87-2042) and there were no differences between subgroups.

**Conclusion(s):**

Patients with pancreatic necrotic collections seem to have a more severe clinical course requiring ICU stay and interventions for drainage of the collection(s). Necrotic collections are more commonly seen in patient with biliary pancreatitis and obesity. No all-cause mortality differences were seen between groups during follow up.

**Please acknowledge all funding agencies by checking the applicable boxes below:**

None

**Disclosure of Interest:**

None Declared

